# Effectiveness of A Respiratory Care Protocol Including Less Invasive Surfactant Administration in ≥ 35 Weeks Gestational Age Infants

**DOI:** 10.1002/ppul.71257

**Published:** 2025-08-14

**Authors:** Etze Chotzoglou, Arun Prasath, Riddhi Desai, Lebanon David, Nancy Ornelas, Patti Burchfield, Larry Steven Brown, David B. Nelson, Venkatakrishna Kakkilaya

**Affiliations:** ^1^ Division of Neonatal‐Perinatal Medicine, Department of Pediatrics University of Texas Southwestern Medical Center Dallas Texas; ^2^ Parkland Health and Hospital System Dallas Texas; ^3^ Department of Obstetrics & Gynecology University of Texas Southwestern Medical Center Dallas Texas

**Keywords:** late preterm, LISA, RDS, surfactant, term

## Abstract

**Background:**

In October 2018, a respiratory care protocol (RCP) including less invasive surfactant administration (LISA), was introduced for preterm infants admitted on continuous positive airway pressure (CPAP).

**Study Design:**

We compared respiratory care practices and outcomes of ≥ 35‐week gestational age (GA) infants between a pre‐RCP (Jan 2016 to September 2018) and a post‐RCP cohort (Oct 2018 to Dec 2021). Infants requiring < 24 h of CPAP and diagnosed with meconium aspiration syndrome were excluded.

**Results:**

Of the 260 infants meeting inclusion criteria, 126 belonged to the pre‐RCP and 134 to post‐RCP cohort. Compared to pre‐RCP, a lower proportion of infants in the post‐RCP received CPAP on admission but a higher proportion received surfactant therapy (8% vs 22%, *p* < 0.001). Notably, surfactant therapy was associated with lower FiO_2_ requirement for 24 h and respiratory severity score for 48 h in the post‐RCP cohort. However, there was no difference in any of the outcomes such as the need for mechanical ventilation, incidence of pneumothorax and length of hospital stay between two cohorts.

**Conclusions:**

Implementing an RCP increased surfactant use with associated improvement in oxygenation but did not improve outcomes. Further studies are necessary to evaluate the role of LISA in ≥ 35‐week GA infants.

## 1 Introduction

Surfactant therapy and continuous positive airway pressure (CPAP) are cornerstones of respiratory support in preterm infants with respiratory distress syndrome (RDS) [1]. Surfactant therapy is also found to decrease mortality and the need for extracorporeal membrane oxygenation (ECMO) in term infants receiving mechanical ventilation (MV) for hypoxic respiratory failure [2, 3]. Traditionally, surfactant was administered via an endotracheal tube (ETT). Recently, less invasive surfactant administration (LISA) using a thin catheter has shown to decrease the need for MV and improve outcomes in preterm infants compared to surfactant administration via ETT [4, 5]. Several guidelines recommend LISA as an early rescue strategy using fraction of inspired oxygen (FiO_2_) 0.3‐0.4 in the treatment of RDS in preterm infants < 30 weeks’ gestational age (GA) [1, 6, 7].

Late preterm infants (34‐36 weeks GA) make up for over 7% of all live births in the United States and represent nearly 75% of all preterm births [8, 9]. In a large cohort study in the United States, Hibbard et al. reported that 36.5% of late preterm infants and 7.2% of term infants were admitted to NICU. The incidence of RDS decreases with increasing GA between 34 and 38‐weeks GA [10]. RDS is the most common reason for MV in late preterm infants while pneumonia, meconium aspiration syndrome (MAS) and persistent pulmonary hypertension of newborn (PPHN) in term infants [11]. Several cohort studies show benefits of surfactant in late preterm and term infants with RDS, MAS and pneumonia [3, 12]. A recent meta‐analysis which included 16 observational studies and a randomized control trial (RCT) showed that surfactant therapy in late preterm and term infants with RDS may decrease mortality, air leak, PPHN and duration of respiratory support [13]. Although the benefits of surfactant are well known, there is no consensus on the threshold for its use in late preterm and term infants with suspected surfactant deficiency or inactivation. Moreover, the benefits of LISA over ETT surfactant administration are not established in this group of infants.

In our center, we implemented a quality improvement respiratory care protocol (RCP) consisting of optimization of CPAP and LISA guided by FiO_2_ ≥ 0.3 starting in October 2018, primarily for infants ≤ 29 weeks’ GA. We reported a decrease in CPAP failure and pneumothorax in ≤ 29 weeks’ GA infants compared to historical controls [14, 15]. The success of the protocol prompted the extension of its use in infants ≥ 30 weeks GA requiring CPAP for respiratory distress. The objective of our study is to evaluate the effects of the RCP in ≥ 35 weeks GA infants. We hypothesized that the establishment of RCP in clinical practice would decrease the need for MV in ≥ 35 weeks GA infants.

## 2 Materials and Methods

### 2.1 Study Design and Study Population

This is a retrospective study of infants ≥ 35 weeks GA, born between January 2016 and December 2021 at Parkland Hospital. Infants admitted on CPAP requiring surfactant therapy or MV were included in the study. Infants with transient respiratory distress defined as need for CPAP for < 24 h were excluded. Infants intubated in the delivery room (DR), infants with MAS, and major congenital anomaly, infants requiring respiratory support for surgery or neurologic conditions were excluded from the study. Infants born between January 2016 and September 2018 were included in the pre‐RCP and those born between October 2018 and December 2021 were included in the post‐RCP cohort.

### 2.2 Setting

Parkland is a large county hospital with over 12 thousand deliveries per annum. All low‐risk deliveries of infants ≥ 35 weeks GA are attended by a Neonatal Resuscitation Program (NRP) trained nurse and pediatric resident or pediatric nurse practitioner. Starting in 2011, as per the 2010 NRP guidelines [16, 17], infants with respiratory distress or cyanosis were treated with CPAP in DR. A protocol change was initiated in April 2017 to limit the use of CPAP in the DR to infants with respiratory distress only when they require supplemental oxygen to achieve NRP specified oxygen saturation goals [18, 19]. CPAP in the DR is started at 5 cm H_2_O using face mask connected to a T‐Piece resuscitator (Neopuff ^TM^, Fisher & Paykel, Auckland, N Z). Infants requiring CPAP for transport were changed to bi‐nasal prongs (Hudson RCI, Medline, IL), connected to a positive end expiratory pressure (PEEP) valve, running 8‐10 liter/minute flow. Cord blood gas was routinely collected on all infants. Arterial blood gas and a chest radiograph are obtained on all infants receiving supplemental oxygen and/or CPAP. Infants born ≥ 35 weeks GA are routinely cared for in the mother‐baby unit, but they are admitted to the NICU if requiring any respiratory support or in need of close monitoring.

As mentioned above, starting October 2018, an RCP was implemented in our center consisting of a stepwise escalation of CPAP from 5 to 7 cm H_2_O followed by LISA guided by FiO_2_ ≥ 0.3. The escalation of CPAP and LISA were done on a timely manner with a set limit of 30 min between each step (supplementary figure 1). The surfactant of choice was Poractant alfa, 200 mg/kg for the initial dose and 100 mg/kg for subsequent doses, for a maximum of three doses.

Before implementation of the RCP, decisions to increase CPAP, intubate, initiate MV and administer surfactant were based on physician's discretion. Generally, surfactant therapy was considered at CPAP 5–7 cm H_2_O and FiO_2_ 0.45–0.5 for infants with RDS. Intubation‐Surfactant‐Extubation (INSURE) was used per physician discretion. Implementation of the RCP was the only clinical practice change occurred in the NICU during the study period. Analgesia or sedation was not routinely used for LISA or endotracheal intubations during the study period.

### 2.3 Data Collection and Definition

Maternal and neonatal baseline characteristics were obtained from the validated Parkland NICU database. Clinical diagnoses for respiratory distress, including RDS, transient tachypnea of the newborn (TTN), pneumonia, and PPHN, were also obtained from the database but confirmed with a retrospective chart review of individual patients. Details of resuscitation at birth and CPAP level and FiO_2_ on NICU admission and at different hours of life were collected. In addition, CPAP level and FiO_2_ before and after surfactant administration were collected to evaluate the changes in the respiratory status in relationship to surfactant administration. Details of cord blood gas and admission arterial blood gas (ABG) values were also collected. The Respiratory Severity Score (RSS = CPAP cm H_2_O * FiO_2_), Alveolar (A)‐arterial (a) PO_2_ gradient [(FiO_2_ *713) ‐ (PaCO_2_/0.8) ‐ PaO_2_], a/A PO_2_ (PaO_2_/[(FiO_2_ * 713) ‐ (PaCO_2_/0.8)]) were calculated.

### 2.4 Statistical Analysis

Maternal and infant demographics, resuscitation details, cord blood gas values, respiratory support on admission at 2 and 4 h of life, arterial blood gas values, surfactant use and administration method, need for MV, duration of respiratory support, length of stay and infant outcomes were compared between the pre‐and post‐RCP cohorts using analysis of variance (ANOVA), Kruskal‐Wallis or Dunn test, two‐sample t‐test, the Mann‐Whitney U test, and the χ2 test, depending on the distribution of variables. Using logistic regression analysis, we analyzed the relationship of the CPAP liberation time between the cohorts of infants receiving surfactant while controlling for the RSS prior surfactant administration. Count and frequency were reported for categorical variables; median and interquartile range (IQR) and standard deviation (SD) were reported depending on the distribution of continuous variables.

### 2.5 Ethical Consideration

The study was approved by the Institutional Review Board of the University of Texas Southwestern Medical Center (#STU‐2021‐0032), and consent for retrospective data collection was waived.

## 3 Results

**Figure 1:**
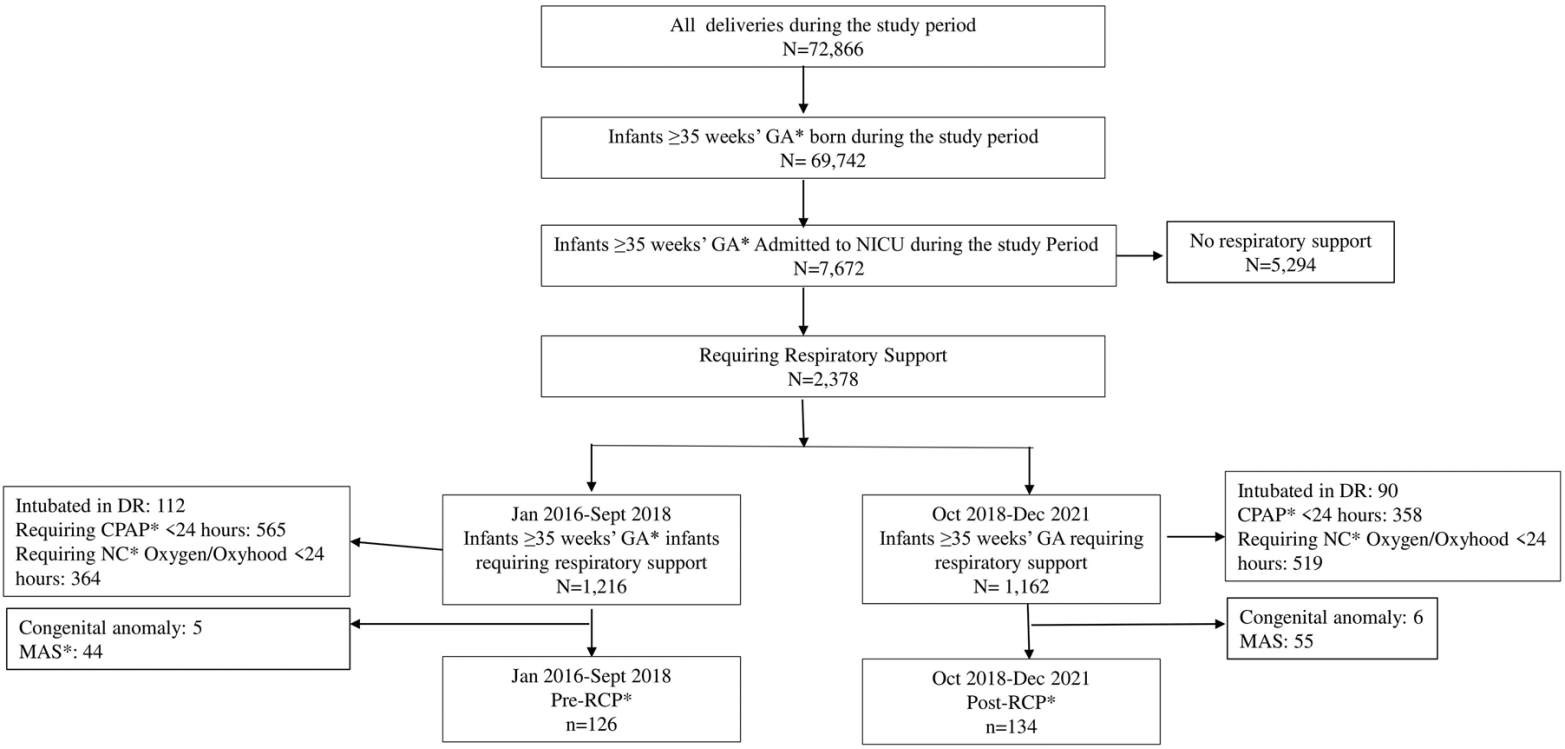
Flow diagram. *Infants ≥ 35 weeks' GA requiring CPAP ≥24 hours or any MV in the NICU during the first 72 hours of life. CPAP, continuous positive airway pressure; DR, delivery room; DR, delivery room; GA, gestational age; NC, nasal cannula; MAS, meconium aspiration syndrome; RCP, respiratory care protocol.

Of all the 72,866 infants born during the study period in our center, 95.7% were ≥ 35 weeks GA. Overall, 7672 (11%) infants ≥ 35 weeks GA were admitted to the NICU, of whom 31% received respiratory support. Of the 260 meeting the inclusion criteria, 126 infants belonged to pre‐RCP and 134 to post‐RCP (Figure 1).

There were no differences in maternal factors such as age, ethnicity, mode of delivery, incidence of clinical chorioamnionitis, and prolonged rupture of membrane between the two groups. Similarly, there were no differences in neonatal characteristics such as GA, birth weight, and Apgar scores between the two groups (Table 1).

**Table 1:** Comparison of perinatal characteristics between Pre‐RCP and Post‐RCP.

	Pre‐RCP (*n* = 126)	Post‐RCP (*n* = 134)	*p* value
Age, (years)^a^	29 (24, 33)	28 (24, 34)	0.87
Hispanic, *n*, (%)	76 (60)	85 (63)	0.73
PIH, *n*, (%)^b^	44 (35)	38 (28)	0.26
Antenatal steroids, *n*, (%)	6 (5)	3 (2)	0.32
Antenatal magnesium, *n*, (%)	40 (32)	31 (23)	0.12
Multiple birth, *n*, (%)	9 (7)	8 (6)	0.70
Chorioamnionitis, *n*, (%)	8 (6)	11 (8)	0.57
Maternal diabetes, *n*, (%)^c^	23 (18)	24 (18)	0.94
Cesarean section, *n*, (%)	87 (69)	87 (65)	0.48
Prolonged ROM ( > 18 h), *n*, (%)	30 (23)	30 (22)	0.79
GA, (weeks)^a^	36 (35, 38)	36 (36, 39)	0.21
Birth weight, (grams)^a^	2803 (2358, 3526)	2928 (2498, 3620)	0.40
Female n, (%)	52 (41)	52 (39)	0.69
Apgar score: 1 min^a^	8 (5, 8)	8 (6, 8)	0.77
Apgar score: 5 min^a^	8 (7,9)	8 (7,9)	0.81
Cord arterial blood gas pH^a^	7.26 (7.19, 7.30)	7.26 (7.21, 7.31)	0.55
Cord arterial blood gas base deficit^a^	−5.4 (−7.8, −3.2)	−5.5 (−7.9, −3.8)	0.45

^a^ Median (25th, 75th).

^b^ Includes gestational hypertension, pre‐eclampsia and eclampsia.

^c^ Includes gestational diabetes.

Abbreviations: GA, gestational age; PIH, pregnancy induced hypertension; RCP: respiratory care protocol, ROM, rupture of membrane.

Furthermore, there were no differences in the proportion of infants requiring positive pressure ventilation (PPV) at birth. However, a lower proportion of infants in the post‐RCP received CPAP in the DR and on admission to NICU. There was no difference in the maximum FiO_2_ and CPAP level in the DR or on admission to NICU between two groups. Notably, a higher proportion of infants in the post‐RCP received surfactant than pre‐RCP cohort (22% vs 8%, *p* = 0.001). There was no significant difference in the time to surfactant therapy between the cohorts. However, infants in the post‐RCP received surfactant at lower median FiO_2_ compared to pre‐RCP (Table 2).

**Table 2:** Comparison of respiratory support between Pre‐ and Post‐RCP cohort.

Characteristics	Pre‐RCP (*n* = 126)	Post‐RCP (*n* = 134)	*p* value
Respiratory support in DR, *n* (%)	101 (80)	97 (72)	0.14
PPV in DR, *n* (%)	41 (33)	33 (25)	0.16
Maximum PIP cm H_2_O^a^	25 (25, 25)	25 (25, 25)	0.48
Maximum FiO_2_ in DR^a^	0.4 (0.3, 0.6)	0.4 (0.21, 0.60)	1.00
CPAP requirement in DR, *n* (%)	84 (67)	69 (52)	0.01
Maximum CPAP cm H_2_O in DR^a^	5 (5, 5)	5 (5, 5)	0.36
Admission FiO_2_ (%)^a^	0.30 (0.21, 0.40)	0.30 (0.21, 0.40)	0.19
Admitted on CPAP, *n* (%)	82 (65)	64 (48)	0.005
Admission CPAP cm H_2_O^a^	5 (5, 5)	5 (5, 5)	0.15
Admission RSS^a^	1.4 (1.05, 1.85)	1.53 (1.25, 2.19)	0.02
Initial arterial blood gas values			
pH^a^	7.28 (7.22, 7.34)	7.27 (7.22, 7.34)	0.68
PaCO_2_ (mmHg)^a^	46 (40, 54)	47 (38, 54)	0.79
PaO_2_ (mmHg)^a^	52 (42, 63)	54 (46, 65)	0.26
Base deficit (mmol/L)^a^	−4.5 (−7.0, −2.0)	−5.0 (−7.0, −3.4)	0.04
Alveolar‐arterial O_2_ gradient^a^	78 (46, 164)	89 (49, 159)	0.43
Arterial‐alveolar ratio	0.39 (0.24, 0.54)	0.36 (0.24, 0.53)	0.59
FiO_2_ at 2 HOL (%)^a^	0.25 (0.21, 0.30)	0.25 (0.21, 0.32)	0.53
CPAP‐at 2 HOL (cmH_2_O)^a^	5 (5, 6)	5 (5, 5)	0.87
RSS at 2 HOL^a^	1.25 (1.05, 1.80)	1.33 (1.05, 1.79)	0.83
RDS, *n* (%)	39 (31)	42 (31)	0.95
TTN, *n* (%)	26 (21)	21 (16)	0.30
Pneumonia, *n* (%)	60 (48)	67 (50)	0.70
PPHN, *n* (%)	15 (12)	12 (9)	0.44
iNO, *n* (%)	4 (3)	2 (2)	0.44
Pre‐surfactant FiO_2_ a	0.55 (0.43, 0.78)	0.40 (0.30, 0.60)	0.05
Pre‐ Surfactant PEEP (cm H_2_O)^a^	6 (6, 7)	7 (6, 7)	0.20
Pre‐Surfactant RSS^a^	3.50 (2.40, 4.95)	2.42 (2.03, 3.79)	0.09
Surfactant treatment *n*, (%)	10 (8)	30 (22)	0.001
LISA *n*, (%)	0 (0)	26 (19)	< 0.01
ETT surfactant *n*, (%)	10 (8)	4 (3)	0.08
Reason for surfactant administration			1.00
RDS *n*, (%)	10/10 (100)	29/30 (97)	
PPHN *n*, (%)	0/10 (0)	1/30 (3)	
Infants with RDS receiving surfactant	9/39 (23)	22/42 (52)	< 0.01
Time to surfactant (hours: min of life)^a^	23:15 (19:26, 28:23)	14:47 (5:21, 27:30)	0.16

^a^ Median (25th, 75th).

Abbreviations: HOL, hours of life; PPV, positive pressure ventilation; RCP, respiratory care protoco; RSS, respiratory severity score.

The FiO_2_ requirement was lower at 2, 4, 12 and 24 h after surfactant therapy in the post‐RCP infants compared to pre‐RCP infants. Similarly, compared to pre‐RCP, the respiratory severity score and CPAP level were lower at 12, 24 and 48 h after surfactant therapy in the post‐RCP. However, there was no statistically significant association between the time to liberation from the CPAP and surfactant administration between the pre‐ and post‐RCP cohorts (Table 3).

**Table 3:** Comparison of response to surfactant between Pre‐ and Post‐RCP cohort.

	Pre‐RCP (*n* = 10)	Post‐RCP (*n* = 30)	*p* value
FiO_2_ 2 h after surfactant^a^	0.37 (0.29, 0.55)	0.25 (0.21, 0.30)	0.006
CPAP 2 h after surfactant (cm H_2_O)^a^	6 (6, 7)	6 (6, 7)	0.76
RSS 2 h after surfactant^a^	1.98 (1.33, 2.45)	1.47 (1.26, 1.56)	0.16
FiO_2_ 4 h after surfactant^a^	0.32 (0.28, 0.55)	0.23 (0.21, 0.30)	0.004
CPAP 4 h after surfactant (cm H_2_O)^a^	6 (5, 7)	6 (5, 6)	0.59
RSS 4 h after surfactant^a^	1.74 (1.21, 2.24)	1.38 (1.05, 1.68)	0.24
FiO_2_ 12 h after surfactant^a^	0.30 (0.24, 0.50)	0.23 (0.21, 0.25)	0.01
CPAP 12 h after surfactant cm H_2_O^a^	7 (6, 7)	6 (5, 6)	0.046
RSS 12 h after surfactant^a^	1.72 (1.37, 2.28)	1.15 (1.05, 1.49)	0.02
FiO_2_ at 24 h after surfactant^a^	0.28 (0.23, 0.78)	0.23 (0.21, 0.25)	0.03
CPAP 24 h after surfactant (cm H_2_O)^a^	7 (6, 7)	5 (4, 6)	0.01
RSS 24 h after surfactant^a^	1.63 (1.48, 1.88)	1.05 (0.84, 1.44)	0.03
FiO_2_ 48 h after surfactant^a^	0.28 (0.21, 0.65)	0.21 (0.21, 0.24)	0.09
CPAP 48 h after surfactant (cm H_2_O)^a^	6 (5, 6)	5 (2, 5)	0.02
RSS 48 h after surfactant^a^	1.26 (1.16, 1.60)	0.99 (0.16, 1.21)	0.02
Liberation from CPAP (hours), adjusted for pre‐surfactant respiratory severity score^a^	120 (90, 174)	96 (72, 120)	0.15

^a^ Median (25th, 75th).

Abbreviations: CPAP, continuous positive airway pressure; FiO_2_, fraction of inspired oxygen; RCP, respiratory care protocol; RSS, respiratory severity score.

There were no significant differences in any of the outcomes such as the need for intubation and MV, duration of MV, length of hospital stay, incidence of pneumothorax, and mortality between the cohorts (Table 4).

**Table 4:** Comparison of outcomes between Pre‐ and Post‐RCP cohort.

	Pre‐RCP (*n* = 126)	Post‐RCP (*n* = 134)	*p* value
Intubated during hospital stay *n*, (%)	17 (14)	11 (8)	0.17
Mechanically ventilated during hospital stay *n*, (%)	12 (10)	11 (8)	0.71
Duration of mechanical ventilation (hours)^a^	52 (0, 101)	50 (20, 87)	0.70
Length of hospital stay (days)^a^	11 (8, 15)	12 (8, 17)	0.09
Pneumothorax *n*, (%)	12 (10)	12 (9)	0.87
Mortality *n*, (%)	0 (0)	0 (0)	N/A

^a^ Median (25th, 75th).

Abbreviation: RCP, respiratory care protocol.

## 4 Discussion

This single‐center study shows that adopting a RCP including stepwise escalation of CPAP and LISA guided by FiO_2_ ≥ 0.3 for ≥ 35 weeks GA infants led to higher use of surfactant and associated improvement in oxygenation. However, RCP did not decrease the need for MV nor improve any other outcomes during the study period. To the best of our knowledge, this is the first study to evaluate the effectiveness of an early rescue LISA guideline in infants ≥ 35 weeks GA.

Our study findings are similar to other studies comparing the use of surfactant in late preterm and term infants. Notably, infants in these studies received surfactant through ETT, while infants in our study received surfactant by LISA method. Of these studies, a multi‐center RCT by Escobedo et al [20], compared between early INSURE and expectant management with CPAP in infants weighing ≥ 1250 grams and ≤ 36 weeks GA. Compared to the control group, infants in the early INSURE group received higher number of surfactant but required a longer duration of MV [20]. Similarly, two prospective observational studies [21, 22] found no difference in either MV requirements or length of hospital stay with surfactant therapy. Additionally, similar to our study results, Dani et al [21] reported improved oxygenation following surfactant therapy. However, our study results are in contrast with a meta‐analysis that reported a decreased risk of mortality, air leak, PPHN, duration of respiratory support, and length of hospital stay in 34‐38 weeks GA infants with RDS [13]. The observed differences may be attributed to variations in the gestational age, interventions and sample sizes between our study and the studies included in this meta‐analysis. Notably, most of the studies included 34–36 weeks GA with RDS while our study included infants ≥ 35 weeks GA demonstrating respiratory distress from various etiology.

There is limited evidence regarding LISA in late preterm and term infants. To date, only two small RCTs compared between LISA and surfactant administration via ETT using different study designs. Our study results are in contrast to the study by Olivier et al [23] that showed lower composite incidence of pneumothorax or need for MV within 72 h of life in the LISA group compared continued CPAP therapy (*n* = 45). However, our results are similar to a study by Yang et al [24] that showed no difference in the need for MV within 72 h of life with LISA compared to INSURE (*n* = 97). Further studies are necessary to evaluate the effectiveness of LISA in late preterm and early term infants.

We included all infants with respiratory distress including from various etiologies. RDS, TTN, and pneumonia can all present similarly with respiratory distress immediately after delivery, making it difficult to differentiate between these diagnoses at that time. Often, these patients are managed similarly in the NICU, with their final diagnoses being confirmed a few days later based on their responses to treatment. Conflicting evidence exists regarding the use of surfactant therapy in infants with respiratory distress other than RDS [3, 25]. While some studies indicate a reduced need for extracorporeal membrane oxygenation (ECMO) after surfactant administration in late preterm or term infants experiencing severe hypoxic respiratory failure due to these conditions [3], there is conflicting evidence regarding short‐term outcomes, such as the duration of hospitalization and oxygen requirement [25]. Currently, a large RCT is enrolling ≤ 24 h old, 34–38 weeks GA infants to compare between early surfactant and expectant management with CPAP alone on the duration of hospital stay and incidence of severe respiratory failure (SURF‐ON study) [26].

The optimal threshold for surfactant therapy in late preterm and term infants is not well established. The American Academy of Pediatrics consensus statement concluded that delaying or withholding surfactant in preterm infants stabilized on CPAP at birth does not lead to adverse outcomes [27]. The recent RDS NExT workshop group also noted this lack of consensus [1]. At our center surfactant was administered based on the FiO_2_ threshold in both cohorts. The RCP encouraged timely escalations of CPAP and LISA guided by FiO_2_ threshold of > 0.3. As expected, infants in the post‐RCP received surfactant at lower FiO_2_ which resulted in increased use of LISA. However, despite adherence to the guideline, there was no significant difference in the time to surfactant therapy between the two groups. This suggests the need for a better tool to identify infants with RDS and other disease processes earlier.

Late preterm infants and early term infants account for 7.7% and 28.8% of all live births [8]. Late preterm infants account for 75% of infants born premature [9]. Decreasing the need for mechanical ventilation, duration of respiratory support and hospital stay can lead to substantial cost savings to health care system [10, 28]. Our study results suggests that a respiratory care guideline including LISA alone may not be enough to impact any of the above cost‐effective metrics in the population studied. A previous study developed a cost‐consequence model to compare early rescue surfactant administration strategies, such as LISA and INSURE, with standard administration via ETT and MV in preterm infants born between 25 and 32 weeks of GA with RDS. This study found that early rescue strategies like LISA and INSURE were associated with a lower number of infants needing MV, a reduced duration of ventilation, and fewer neonatal complications. Although the total annual costs of surfactant were higher with these early rescue strategies, the savings in overall hospital and complication costs offset this increase, suggesting a lower overall healthcare burden with early rescue methods [29]. Further studies are needed to identify late preterm infants who might benefit from surfactant administration, which could help enhance the cost–benefit ratio of this intervention in this specific group of infants.

This study has several strengths. First, it is the first study to evaluate the benefits of a noninvasive respiratory support guideline incorporating LISA in ≥ 35 weeks GA infants with respiratory distress. Although over 40% of late preterm and early term infants with RDS receive surfactant therapy [21], there is a paucity of evidence on the benefits of a less invasive method of surfactant administration and the optimum threshold. This study shows that a lower threshold for surfactant administration may be associated with higher use of surfactant. The findings of this study can help design prospective studies to target surfactant therapy for infants at risk of adverse outcomes such as pneumothorax and mechanical ventilation. Second, we included all ≥ 35‐week‐old GA infants requiring significant respiratory support for various respiratory illnesses without limiting to infants with a radiological diagnosis of RDS. This reflects the real‐life situation given the uncertainty of chest radiographic interpretation in diagnosing the cause of respiratory distress in infants. Third, our study used the data from the validated databases, maintaining uniformity with the diagnosis of different clinical conditions over an extended time period. Fourth, the group of infants included in the study is drawn from a large cohort of infants delivered in a single center. The proportion of infants delivered at ≥ 35 weeks GA is similar to the National Vital Statistics report [9]. Similarly, the proportion of infants admitted to NICU for respiratory illness is similar to data from a large multicenter cohort. Moreover, infants admitted to the NICU for respiratory distress throughout the study period are initially managed using guidelines defining the role of the nasal cannula and CPAP, limiting variations in practice between physicians in selecting the initial support modality.

Our study has several limitations. First, this is a retrospective study spanning over 5 years. Several co‐interventions could impact the study outcome. A lower proportion of infants received CPAP in DR and on admission, which may be related to the changes in the guideline for CPAP use in DR. Second, compliance with the RCP was not monitored during the study period. However, higher use of surfactant at lower FiO_2_ levels suggests that there was adherence to the protocol. Furthermore, in the entire post‐RCP cohort, four infants received surfactant via ETT following provider‐dependent criteria for surfactant therapy. Third, we have not collected information about the procedure. This information would have provided additional data on procedural safety. Fourth, this is a single‐center study done in a tertiary‐level teaching hospital. Caution should be applied while generalizing the findings of our study.

In conclusion, our single‐center retrospective study shows that implementation of a respiratory care protocol consisting of optimization of CPAP and LISA guided by FiO2 ≥ 0.3 is associated with higher use of surfactant in late preterm infants. Although higher surfactant use was associated with improved oxygenation there was no reduction in the need for MV or improvement in any other outcomes, such as duration of hospital stay and pneumothorax rates. Though a subset of late preterm and term infants might benefit from surfactant, future studies should focus on carefully selecting these patients using precise clinical and possibly radiological parameters. This approach may prevent excessive use of surfactant, thereby reducing associated costs and resource utilization while offering limited additional benefits. Further adequately powered studies are necessary to evaluate the benefits of a combined strategy of optimization of CPAP and LISA in ≥ 35 weeks GA infants.

## Author Contributions

**Etze Chotzoglou:** conceptualization, writing – original draft, methodology, writing – review and editing, investigation, data curation. **Arun Prasath:** writing – review and editing, methodology. **Riddhi Desai:** writing – review and editing, methodology. **Lebanon David:** writing – review and editing, data curation. **Nancy Ornelas:** writing – review and editing, data curation. **Patti Burchfield:** writing – review and editing, data curation. **Larry Steven Brown:** writing – review and editing, software, formal analysis, methodology, validation. **David B Nelson:** methodology, writing – review and editing. **Venkatakrishna Kakkilaya:** writing – review and editing, validation, methodology, supervision, resources, investigation, formal analysis.

## Consent

The authors have nothing to report.

## Conflicts of Interest

The authors declare no conflicts of interest.

## Supporting information

Supporting Figure 1. Respiratory Care Protocol (RCP).

## Data Availability

The data that support the findings of this study are available on request from the corresponding author. The data are not publicly available due to privacy or ethical restrictions.
