# Supplementary figures and images for: Effectiveness of A Respiratory Care Protocol Including Less Invasive Surfactant Administration in ≥ 35 Weeks Gestational Age Infants
Source: Pediatr Pulmonol. 2025 Aug 14;60(8):e71257. doi: 10.1002/ppul.71257 (PMC12352716; doi:10.1002/ppul.71257)

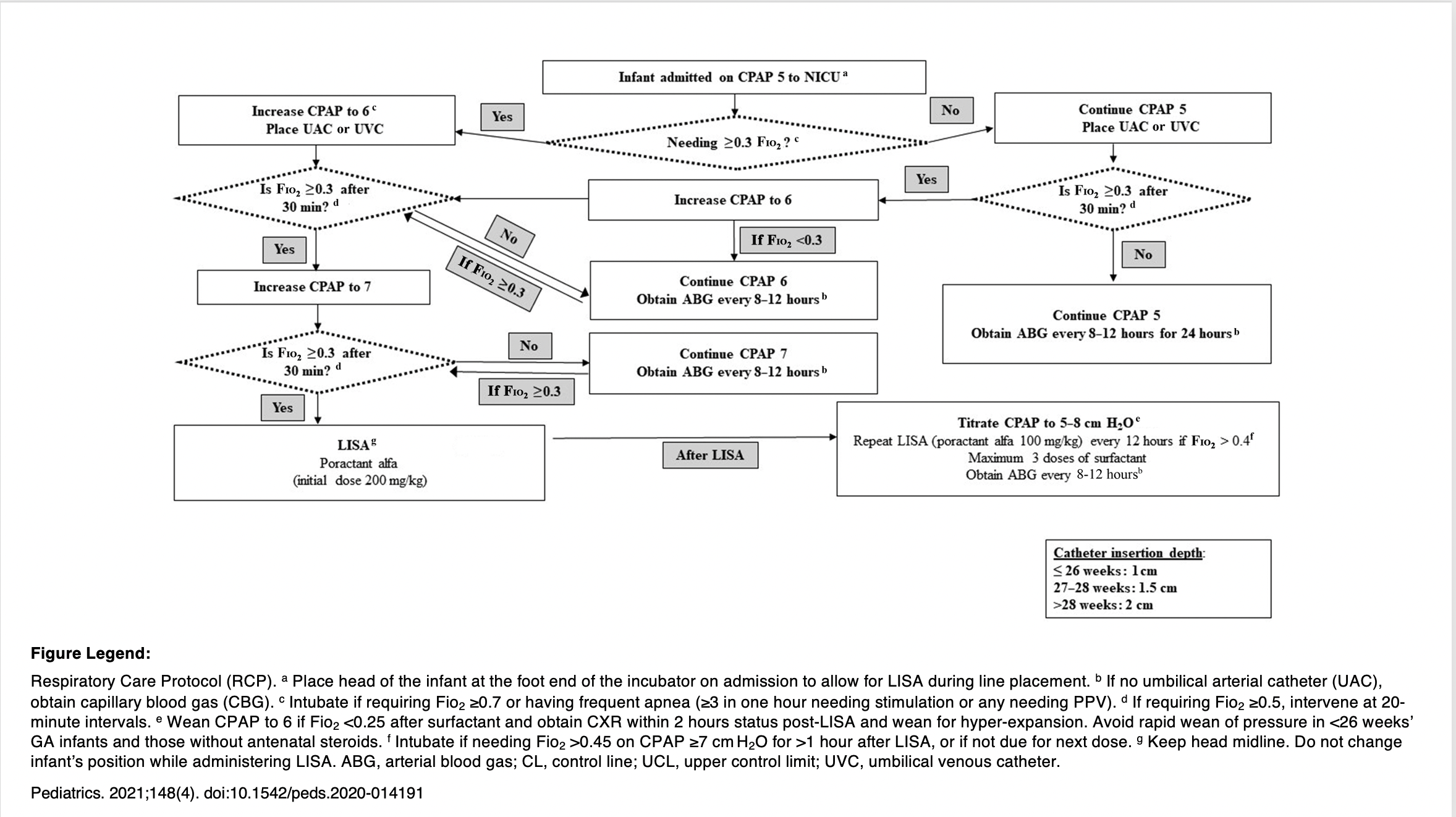

Supplement: Supplementary file 1 — Supporting Figure 1. Respiratory Care Protocol (RCP). [file PPUL-60-0-s001.png]
